# *FOXE1* polymorphism rs965513 predisposes to thyroid cancer in a European cohort

**DOI:** 10.1530/EO-21-0003

**Published:** 2021-05-19

**Authors:** Patrick W Owens, Terri Patricia McVeigh, Nicola Miller, Carole Guerin, Frederic Sebag, Denis Quill, Marcia Bell, Michael J Kerin, Aoife J Lowery

**Affiliations:** 1 Discipline of Surgery, Lambe Institute for Translational Research, School of Medicine, National University of Ireland, Galway, Ireland; 2 Cancer Genetics Unit Royal Marsden NHS Foundation Trust, London, England; 3 Department of Endocrine Surgery Centre hospitalo-universitaire de La Conception, Assistance Publique Hôpitaux de Marseille, Marseille, France; 4 Department of Endocrinology, School of Medicine, National University of Ireland, Galway, Ireland

**Keywords:** differentiated thyroid cancer, gene polymorphism, genetics, risk assessment, prognostication

## Abstract

**Objective:**

*FOXE1* is an intronless gene on chromosome 9 which plays a significant role in thyroid morphogenesis. Mutations in *FOXE1* are associated with thyroid phenotypes including congenital hypothyroidism, thyroid dysgenesis and thyroid cancer. This study aims to investigate the frequency and impact of a SNP (rs965513, G>A) at 9q22.23 in a Western European cohort of patients with differentiated thyroid cancer(DTC), compared to controls.

**Design:**

This is a candidate gene case control study.

**Methods:**

277 patients with histologically confirmed DTC were recruited from tertiary referral centres in Ireland and France. 309 cancer-free controls were recruited from the community. DNA was extracted from buccal swabs or whole blood of control subjects and patients with DTC. Allelic and genotypic frequencies among patients were compared with controls, to assess the risk for disease conferred by homozygous and heterozygous carriers compared to WT genotypes. Genotyping was performed using Taqman-based PCR.

**Results:**

277 patients with confirmed DTC and 309 non-cancer controls were genotyped for the variant (rs965513). The frequency of the minor allele among cases was 0.45 compared to 0.34 among controls. The genotypic odds ratio for heterozygotes was 1.66 (CI 1.16–2.39, *P* =0.00555), increasing to 2.93 (CI 1.70–5.05, *P* =0.00007) for rare homozygotes. All subjects were in Hardy-Weinberg equilibrium (±*χ*^2^, *P* =0.09, *P* =0.07 respectively).

**Conclusions:**

This *FOXE1* polymorphism is a low penetrance variant associated with DTC susceptibility in this cohort. The minor allele was identified among patients with thyroid cancer significantly more frequently than controls. An allele dosage effect was observed, with rare homozygous genotypes conferring greater risk than heterozygotes.

## Introduction

Thyroid cancer is the most common endocrine malignancy with an increasing incidence recorded over recent decades in Western countries, including Ireland. Genetic factors play a significant role in the determination of thyroid cancer risk (Amundadottir *et al.* 2004). Epidemiological evidence arising from the Swedish Family-Cancer Database of 9.6 million people, estimates that 53% of thyroid cancer risk (all subtypes) is accounted for by genetic rather than environmental factors - the highest among all cancers studied (Czene *et al.* 2002). Medullary thyroid cancer development is associated with point mutations in the RET proto-oncogene on chromosome 10, of which approximately 25% are germline mutations (Accardo *et al.* 2017). While less is known about the sequence variations which confer an increased risk for differentiated thyroid cancer (DTC), it is unlikely that a similar high risk, high penetrance mutation will be found to underlie its pathogenesis. Notwithstanding this, 5–15% of non-medullary thyroid cancer cases are thought to be of familial origin (Khan *et al.* 2010). Multiple mutations, each with relatively smaller effect sizes, together with environmental factors, are more likely to give rise to the majority of DTC (Saenko & Rogounovitch 2018). Numerous genetic alterations have thus far been identified to play a role in thyroid tumourigenesis. Better known examples include mutations in the mitogen-activated protein kinase (MAPK) pathway. The most common of these include BRAF V600E mutations in up to 60% of PTCs, RAS mutations in 15% of PTCs and RET/PTC, ALK or NTRK1 chromosomal rearrangements in 12% of papillary carcinomas (Fagin & Wells 2016, Poller & Glaysher 2017). Other implicated co-existing mutations include TERT promoter, PIK3CA, AKT1 and TP53 mutations (Xing 2013).

Single nucleotide polymorphisms (SNPs) form the basis of many genetic mutations and arise due to nucleotide variation at a single position in a DNA sequence. Genome-wide association studies (GWASs) have identified numerous candidate SNPs which confer increased risk for the development of DTC. Due to the geographical heterogeneity of DTC incidence and pathology, single-track candidate gene studies have subsequently been required to validate candidate SNPs identified by GWAS in various other populations. One such SNP (rs965513, G>A) is located on 9q22.33, at the *FOXE1* gene. The intronless *FOXE1* gene encodes forkhead box protein E1, belonging to the forkhead family of transcription factors and is important for initiation of thyroid organogenesis, cell growth and growth-factor control of thyroid differentiation. *FOXE1* mutations have been associated with phenotypes including congenital hypothyroidism and thyroid dysgenesis. *FOXE1* plays a role in thyroid tumour development, invasion and metastases, is a gene of interest in thyroid cancer research with multiple thyroid cancer risk SNPs near *FOXE1* recently identified (Gudmundsson *et al.* 2009, Chen & Zhang 2018).

This study investigates the impact of a SNP, rs965513 at the *FOXE1* locus, on predisposition to differentiated thyroid cancer in a Western European patient population.

## Materials and methods

A casecontrol methodology was utilised to assess the impact of the candidate gene mutation (rs965513, G>A) on DTC risk. Allelic and genotypic frequencies among 277 patients with confirmed DTC were compared with 309 non-cancer controls, to assess DTC risk conferred by homozygous and heterozygous carriers compared to WT genotypes.

A thyroid cancer Biobank was first established at the Discipline of Surgery in the Lambe Institute for Translational Research as part of a multi-centre initiative, comprising clinicopathological data and tissue from patients attending endocrine cancer clinics at tertiary referral centres in the West of Ireland (Galway University Hospital) and in the South of France (Assistance Publique Hôpitaux de Marseille). Patients with histopathologically confirmed DTC, >16 years old, were included. Patients with benign thyroid disease alone, medullary thyroid cancer or familial nonmedullary thyroid cancer were excluded. Patients with any known diagnosis of high-risk germline mutations were also excluded. Controls comprised volunteers >60 years old, without a personal or first degree family history of malignancy, excluding non-melanomatous skin cancer.

All participants provided informed written consent before inclusion. Ethics approval was granted following review by the local institutional Research Ethics Committee.

Demographic and clinicopathological data was recorded at the time of tissue collection from patient self-reporting, electronic histopathology database and patient records. Samples comprised either 10 mL EDTA stabilised whole blood or buccal swab salivary sample (DNA Genotek Oragene 575 collection kit).

DNA was extracted by manual ethanol precipitation. Following extraction, evaluation of the DNA concentration and purity was performed with absorbance spectroscopy (NanoDrop 1000 Spectrophotometer). Samples with a 260:280 nm (A260/280) absorbance ratio ≈ 1.8 were accepted as pure DNA. Real-time PCR and genotyping experiments were undertaken using either the Applied Biosystems StepOnePlus Real-Time PCR System or the 7900HT Fast Real-Time PCR System.

Data recording and statistical analysis were performed using Microsoft Excel 2010 and IBM SPSS v22. Parametric tests and means ± standard deviation were utilised for normally distributed data while non-parametric tests and medians with range and interquartile range (IQR) were utilised for non-normally distributed data. Pearson's chi-squared tests were used to compare distributions of categorical variables, while one way ANOVA with Tukey* post-hoc* analysis was utilised for comparisons involving three or more categorical, independent samples. Hardy-Weinberg equilibrium testing of biallelic SNPs was performed using the Pearson's chi-squared test. Chi-squared tests were also used for case-control genetic association analysis.

## Results

277 patients with confirmed DTC and 309 non-cancer controls were genotyped for the variant (rs965513). All patients and controls were observed to be in Hardy-Weinberg equilibrium (*χ*^2^, *P* =0.09, *P* =0.07 respectively). Patient demographics and clinicopathological characteristics are detailed in Tables 1 and 2 below.

**Table 1:** Patient and control demographics.

	DTC cases n (%)	Non-cancer controls n (%)
Patients	277 (100%)	309 (100%)
Gender		
Female	208 (75%)	252 (82%)
Male	69 (25%)	57 (18%)
European Caucasian:	255 (92%)	309 (100%)
Other	22 (8%)	0 (0%)
Age, at diagnosis / sampling		
Mean (± s.d.)	46.9 (± 14.95)	73.4 (± 8.68)
Median (range, IQR)	45 (16–84, 23)	74 (60–100, 10)

**Table 2:** Clinicopathological patient and tumour characteristics.

	DTC cases n (%)
Patients	277 (100)
Tumour size	
Mean (± s.d.)	26 (± 18.05)
Median (range, IQR) (mm)	22 (1–110, 22)
Papillary	236 (85%)
Follicular	41 (15%)
Multi-focal	89 (32%)
Node positive	56 (20%)
Distant metastatic disease	8 (3%)
Lymphovascular invasion	44 (16%)
Personal history of other cancer^1^	25 (9%)
Family history of DTC^2^	23 (8%)

^1^ Excluding non-melanomatous skin cancer; ^2^First degree relatives only.

The minor allele frequency was higher in patients with DTC (0.45) compared to controls (0.34) and conferred an increased risk for DTC (per-allele OR 1.61, CI 1.27–2.04, *P* =0.00008) (Table 3). Both variant genotypes had an increased risk for DTC and demonstrated an allele-dosage association; heterozygous genotypes (AG) had an odds ratio of 1.66 (CI 1.16–2.39, *P* =0.00555) when compared with wild-types states (GG), increasing to 2.93 (CI 1.70–5.05, *P* =0.00007) for rare homozygotes (AA).

**Table 3:** Genotype frequencies, genotypic odds ratios, and minor allele frequencies in DTC for all cases vs controls.

rs965513 (GA)	Common homozygote (GG), n (%)	Heterozygote (AG), n (%)	Rare homozygote (AA), n (%)	Minor allele frequencies
Cancers	77 (28%)	151 (55%)	49 (17%)	0.45
Controls	129 (42%)	152 (49%)	28 (9%)	0.34
Odds ratio (95% CI)		1.66 (1.16–2.39)	2.93 (1.7–5.05)	
Significance (*P*)		0.00555	0.00007	

**Figure 1:**
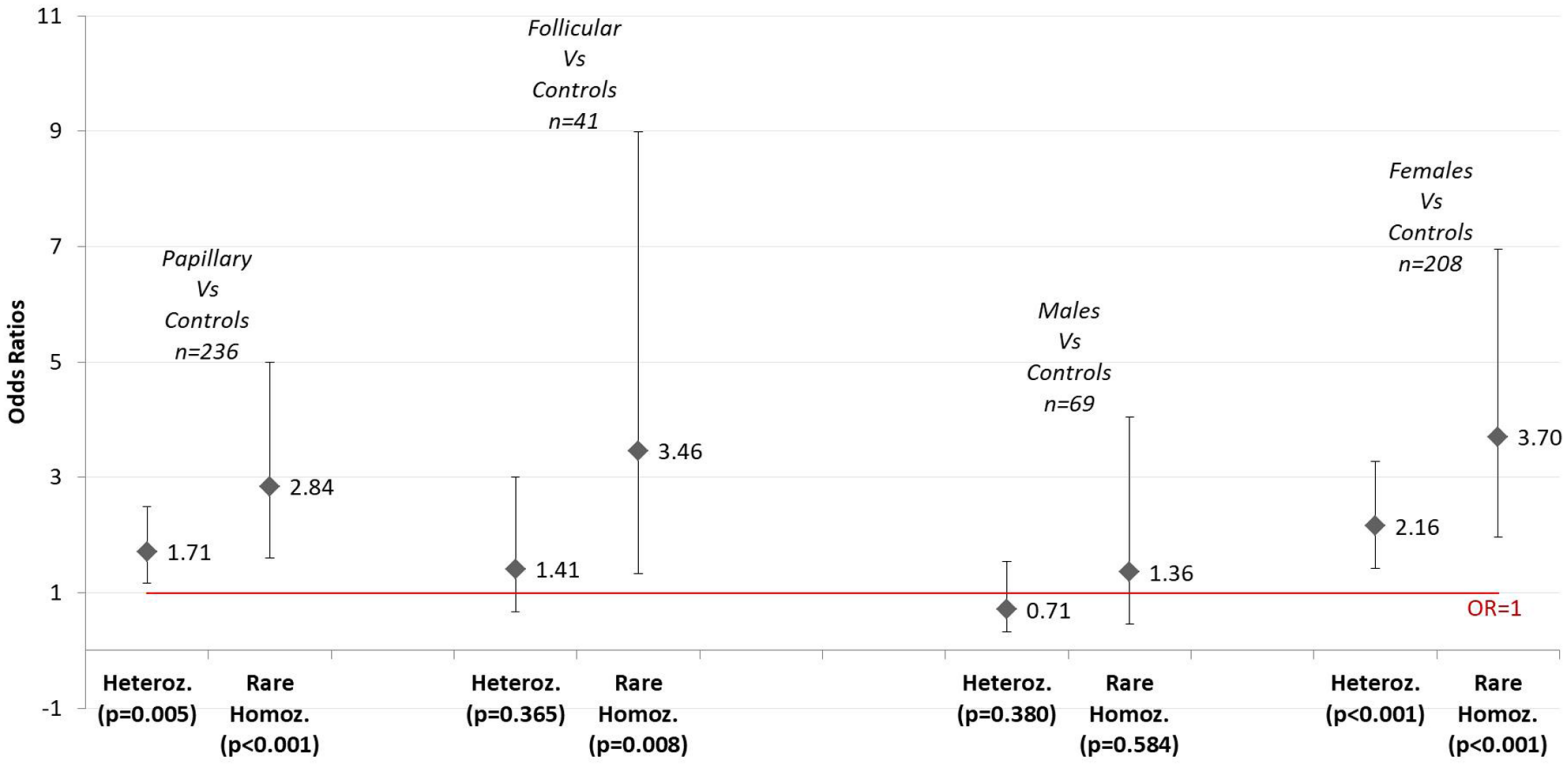
Genotypic odds ratios for sub-groups vs controls comparing rare homozygotes and heterozygotes with wild-types states: histological subtypes and gender.

**Figure 2:**
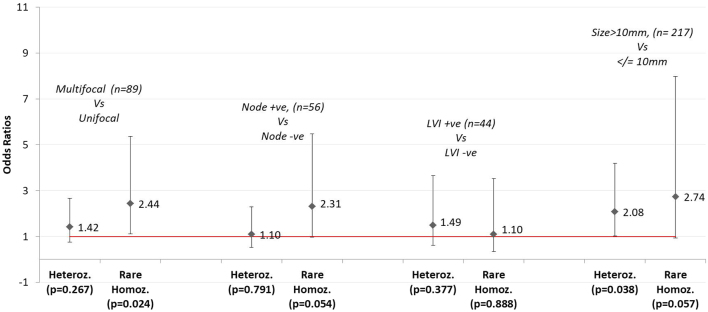
Genotypic odds ratios comparing rare homozygotes and heterozygotes with wild-types states for multi-focality, nodal status, lymphovascular invasion status and tumour size.

Subgroup analysis demonstrated statistically significant associations between the both variant genotypes (AG, AA) and DTC, for female gender but not for males. DTC risk association was also present for rare homozygotes, rather than heterozygous states, for tumours >10 mm, but not for patients with sub-centimetre DTC (Figs 1 and 2 & Table 4).

**Table 4:** Subgroup analysis: genotypic odds ratios and minor allele frequencies.

SNP G>A	Wild-type (GG)	Heterozygote (AG)	Rare homozygote (AA)	Minor allele frequency
Papillary cases	65	131	40	0.48
All controls	129	152	28	0.34
Odds ratio (CI)		1.71 (95% CI 1.17–2.5)	2.84 (95% CI 1.61–5.0)	
*P* value		0.00531210	0.00024632	
Follicular cases	12	20	9	0.46
All controls	129	152	28	0.34
Odds ratio (CI)		1.41 (95% CI 0.67–3.0)	3.46 (95% CI 1.33–8.99)	
*P* value		0.36518017	0.00797090	
Male cases	26	30	13	0.41
Male controls	19	31	7	0.39
Odds ratio (CI)		0.71 (95% CI 0.33–1.54)	1.36 (95% CI 0.46–4.05)	
*P* value		0.38021705	0.58388242	
Female cases	51	121	36	0.46
Female controls	110	121	21	0.32
Odds ratio (CI)		2.16 (95% CI 1.42–3.27)	3.7 (95% CI 1.96–6.96)	
*P* value		0.00026969	0.00003028	
Multi-focal cases	19	49	21	0.51
Unifocal cases	53	96	24	0.42
Odds ratio (CI)		1.42 (95% CI 0.76–2.67)	2.44 (95% CI 1.11–5.36)	
*P* value		0.26740704	0.02448424	
Node +ve cases	13	28	15	0.52
Node -ve cases	60	117	30	0.43
Odds ratio (CI)		1.1 (95% CI 0.53–2.29)	2.31 (95% CI 0.97–5.47)	
*P* value		0.79133678	0.05408739	
LVI* cases	9	28	7	0.48
Non LVI cases	24	50	17	0.46
Odds ratio (CI)		1.49 (95% CI 0.61–3.65)	1.1 (95% CI 0.34–3.53)	
*P* value		0.37714114	0.88753708	
>10mm cases	54	124	39	0.47
≤10mm cases	19	21	5	0.34
Odds ratio (CI)		2.08 (95% CI 1.03–4.18)	2.74 (95% CI 0.94–7.98)	
*P* value		0.03766692	0.05708887	

Variations in MAF and genotypic odds ratios were also observed between Irish and French subgroups, with the minor allele observed more frequently among patients recruited in Ireland, in addition to conferring a greater risk for DTC compared with the French cohort (Table 5). In addition to higher genotypic odds ratios among the Irish subgroup, an increased per-allele ratio of 1.84 (95% CI 1.40–2.41, *P* =0.00001) was also observed, compared with a per-allele odds ratio of 1.31 among the French subgroup (95% CI 0.95–1.80, *P* = 0.09678).

**Table 5:** Genotype frequencies, genotypic odds ratios, and minor allele frequencies in DTC for French and Irish populations vs controls.

rs965513 (G → A)	Common homozygote (GG), n(%)	Heterozygote (AG), n(%)	Rare homozygote (AA), n(%)	Minor allele frequencies
**Controls**	**129 (42%)**	**152 (49%)**	**28 (9%)**	**0.34**
**Irish cases**	**39 (23%)**	**96 (57%)**	**33 (20%)**	**0.48**
**Odds ratio (95% CI)**		2.09 (1.35–3.24)	3.898 (2.10–7.23)	
		*P* = 0.00093	*P*< 0.00001	
**French cases**	**38 (35%)**	**55 (50%)**	**16 (15%)**	**0.40**
**Odds ratio (95% CI)**		1.228 (0.76–1.98) *P* = 0.39606	1.94 (0.95–3.96) *P* = 0.06571	
	

## Discussion

Molecular biomarkers have the potential to improve our estimation of DTC risk, and possess a number of advantages over conventional biomarkers. In particular, germline mutations remain stable irrespective of patient, disease or treatment factors, are measurable at or before disease onset, and are amenable to high-throughput assays, now commonly available in modern laboratories. In light of DTC-specific 5 year survival rates in excess of 95%, and increasing incidence rates worldwide, clinicians are challenged to avoid overdiagnosing and overtreating potentially low grade, indolent thyroid lesions, while appropriately managing those requiring more aggressive treatment. Our results demonstrate an association between the rs965513 SNP in the *FOXE1* gene, and an increased risk for development of DTC. Furthermore, we demonstrate an association with disease phenotype and therefore, this marker may confer prognostic benefits to improve DTC risk stratification.

The rs965513 SNP was initially identified as a risk factor for DTC (OR = 1.75, *P* <0.001) in an Icelandic genome-wide association (Gudmundsson *et al.* 2009). A number of studies have subsequently examined the association in various populations, including two large meta-analyses, which both supported an association between rs965513 and DTC susceptibility, but reported widespread heterogeneity between study methodologies and outcomes, in particular among studies examining Ccaucasian populations (Wang *et al.* 2016, Chen & Zhang 2018). Furthermore, a number of studies have also reported an absence of association between DTC and the risk allele (Denny *et al.* 2011, Kang *et al.* 2014).

To date, just one study has examined the association between rs965513 and DTC susceptibility in a UK population, while no published work has included data from Irish or French cohorts (Jones *et al.* 2012). Incidence of thyroid cancer varies considerably between populations. Amongst European nations, the highest rates of thyroid cancer are seen in Lithuania (15.5/100,000), Italy (13.5/100,000) and France (11.7/100,000) (European Network of Cancer Registries 2017). The incidence in Ireland is 5.8/100,000 (Irish Cancer Society 2017). Furthermore, the rate of change in incidence also varies geographically; for example in France incidence has risen from 3.4 to 11.7 per 100,000 from 1983 to 2002, while in Sweden incidence has remained more stable, reported as 2.4/100,000 in 1958 and 3.5/100,000 in 2002 (Brito *et al.* 2014). In Ireland, incidence has increased three-fold, from 1.9/100,000 in 1994 to 5.8/100,000 in 2017 (National Cancer Registry Ireland 2012, Irish Cancer Society 2017). While the cause of such geographical variation in incidence may be explained by differences in population genetics, other factors likely play a role; these include variable exposure to environmental ionising radiation, and in particular, variations in the prevailing approach to the diagnosis and management of thyroid disease and nodules, with more aggressive investigation, and perhaps over-diagnosis, of thyroid disease playing a role. Despite this, such significant geographical variation in thyroid cancer epidemiology, and reported population-based heterogeneity in DTC risk associated with rs965513, further validation of this risk allele is warranted.

An allele dose association between the rs965513 variant allele and DTC was demonstrated in this study, with odds ratios of 1.66 and 2.93 for heterozygous and rare homozygous states respectively and a per-allele odds ratio of 1.61 compared to controls. This compares to per-allele odds ratios of 1.99, 1.75 and 1.82 reported in UK (*n* =781), Icelandic (*n* =192) and Polish (*n* =1795) cohorts respectively, while one Portuguese study reported a per-allele odds ratio of 2.81 in a group of 80 patients with sporadic non-medullary thyroid cancer (Gudmundsson *et al.* 2009, Jones *et al.* 2012, Tomaz *et al.* 2012, Liyanarachchi *et al.* 2013). The frequency of the minor allele was 0.45 in our cohort which compares to 0.49, 0.49, 0.46 and 0.61 in the UK, Icelandic, Polish and Portuguese cohorts respectively. Variations in risk between populations and subgroups may arise for various reasons including true biological differences, variability in modifiable environmental risk factors or differences in sample size and study methodology.

This study recruited patients from tertiary centres in France (*n* =109) and Ireland (*n* =168). The risk allele was more prevalent in Irish than French subjects (MAF Irish 0.48, French 0.4 and Control group 0.34), with associated higher per-allele odds ratios evident in the Irish subgroup, compared with the French (OR=1.31, *P* =0.096 vs OR=1.84, *P* =0.00001 respectively). Presence of the rare homozygous genotype among French subjects conferred an odds ratio for DTC of 1.94 (*P* =0.066). Rare homozygous genotypes in the Irish subgroup demonstrated an odds ratio of 3.9 (*P* <0.00001); an almost four fold increase in DTC susceptibility in the presence of two variant alleles at the locus represents a significant risk factor for DTC. While our results suggest its impact on DTC risk may be more significant in Irish rather than French patients, it is notable that only 15% (*n* =16/109) of French subjects were rare homozygous carriers, compared to 20% of Irish subjects (*n* =33/168); however, assessment of a larger French cohort may also yield a statistically significant odds ratio for DTC. Perhaps the increased rates of homozygous carriers and the higher MAF amongst Irish DTC patients may be attributable to the relative homogeneity of Irish population genetics, arising due to their island location, geographically isolated from continental Europe, historically low rates of inward migration and the relatively late human colonisation of Ireland compared to the rest of Europe (O'Dushlaine *et al.* 2010). Furthermore, a limitation of this study is the absence of French subjects included in the control arm, which may limit conclusions that can be drawn from subgroup analysis specific to the French subgroup. The observed difference in MAF between populations merits further investigation in a larger study with controls of matched ethnicity.

The minor allele frequency measured 0.46 in female cases and 0.32 in female controls, compared to a narrower differential between male cases and controls of 0.41 and 0.39 respectively. Our cohort exhibited an allele dose gender association, with female risk genotypes carrying a higher risk for DTC compared to female controls, while no significant association was demonstrated between male variant genotypes vs male controls. Heterozygous females carried an odds ratio of 2.6 (95% CI 1.42–3.27, *P* =0.0003), rising to 3.7 (95% CI 1.96–6.96, *P* =0.00003) for rare homozygous females. In contrast, equivalent odds ratios for male variant genotypes vs controls measured 0.71 and 1.36 (*P* =ns) for heterozygotes and rare homozygous carriers respectively. The gender distribution of DTC gives rise to insufficient power to adequately assess the variant effect in our male cases (*n* =69, 25%). However, given the gender differential in MAF, in addition to significant odds ratios observed for female genotypes, a gender variance is evident. Of the above mentioned studies, Liyanarachchi *et al* comment explicitly on gender specific risk conferred by the variant; of their 1795 Polish DTC cases, no difference in MAF or DTC risk was evident between genders. Similarly, Gudmundsson* et al.* reported an absence of significant MAF or rs965513 risk difference between genders in their Icelandic cohort. While our cohort does exhibit an allele dose gender association, these results should be interpreted with caution given that secondary analyses with small sample sizes are at risk of Type 1 error, particularly in light of the above mentioned larger studies provided conflicting results.

In addition to contributing to risk estimation for the initial development of DTC, germline SNPs identified by GWAS or candidate gene studies may also impact the clinical course of DTC after diagnosis, and estimate risk for the development of particular pathological characteristics. Our data suggests that rs965513 is associated with tumours >10mm and multifocality. A recent review identifies rs965513 as being associated with increased DTC tumour size and extra-thyroidal extension (Jendrzejewski *et al.* 2019). Other germline mutations have also been linked to additional pathological characteristics including nodal disease burden, metastatic disease and disease-specific mortality (Jendrzejewski *et al.* 2019). It follows that testing for rs965513 as part of a multigene mutational panel may not only estimate the likelihood of DTC occurrence, but also aid in stratifying patients in terms of locoregional, metastatic or recurrent disease risk, thereby informing decisions around treatment strategies such as the need for completion thyroidectomy, nodal dissection, radioiodine remnant ablation or TSH suppression. Furthermore, genotyping patients for rs965513 potentially adds valuable information for the management of cases that fall into an indeterminate or 'individualised decision making' group as described in the British Thyroid Association guidelines, which are currently based only on standard clinicopathologic features (Perros *et al.* 2014).

Our data is concordant with findings of published GWAS and meta-analyses confirming that rs965513 is a low penetrance variant associated with DTC susceptibility. We conclude that assessment of rs965513 may be considered as part of a gene mutation panel to improve DTC risk stratification in patients with this common malignancy.

## Declaration of interest

The authors declare that there is no conflict of interest that could be perceived as prejudicing the impartiality of the research reported.

## Funding

Dr Patrick Owens was supported by an MD/PhD scholarship from Breast Cancer Research. Dr Terri McVeigh was supported by the Health Researchhttp://dx.doi.org/10.13039/100005622 Board/Health Service Executivehttp://dx.doi.org/10.13039/100018270 National Academic Specialist Registrar Fellowship (NSAFP/2014/1).

## Statement of ethics

All subjects involved in this study provided written informed consent for inclusion and the study protocol was approved by the Institutional Ethics Review Board at Galway University Hospital (Ref 45/05 C.A.151).

## Author contribution statement

AL, MK and FS were the supervising physicians, involved in planning, and supervision of the work. PO, TM, CG, MB and DQ undertook collection of patient samples. PO processed samples and data, performed experiments, and drafted the manuscript. All authors considered the outcomes and reviewed the manuscript.

## References

[bib1] Accardo G Conzo G Esposito D Gambardella C Mazzella M Castaldo F Di Donna C Polisten A Avenia N Colantuoni V, *et al*. 2017 Genetics of medullary thyroid cancer: an overview. International Journal of Surgery 41 S2–S6. (10.1016/j.ijsu.2017.02.064) 28506408

[bib2] Amundadottir LT Thorvaldsson S Gudbjartsson DF Sulem P Kristjansson K Arnason S Gulcher JR Bjornsson J Kong A Thorsteinsdottir U, *et al*. 2004 Cancer as a complex phenotype: pattern of cancer distribution within and beyond the nuclear family. PLoS Medicine 1 e65. (10.1371/journal.pmed.0010065) PMC539051 15630470

[bib3] Brito JP Hay ID Morris JC Low risk papillary thyroid cancer. BMJ 348 g3045. (10.1136/bmj.g3045) 24935445

[bib4] Chen YH Zhang YQ. 2018 Exploration of the association between FOXE1 gene polymorphism and differentiated thyroid cancer: a meta-analysis. BMC Medical Genetics 19 83. (10.1186/s12881-018-0604-y) PMC5964894 29788924

[bib5] Czene K Lichtenstein P Hemminki K Environmental and heritable causes of cancer among 9.6 million individuals in the Swedish Family-Cancer Database. International Journal of Cancer 99 260–266. (10.1002/ijc.10332) 11979442

[bib6] Denny JC Crawford DC Ritchie MD Bielinski SJ Basford MA Bradford Y Chai HS Bastarache L Zuvich R Peissig P, *et al*. 2011 Variants near FOXE1 are associated with hypothyroidism and other thyroid conditions: using electronic medical records for genome- and phenome-wide studies. American Journal of Human Genetics 89 529–542. (10.1016/j.ajhg.2011.09.008) 21981779 PMC3188836

[bib7] European Network of Cancer Registries. Thyroid cancer factsheet. Ispra, Italy: ENCR, 2017. (available at: https://encr.eu/sites/default/files/factsheets/ENCR_Factsheet_Thyroid_2017-2.pdf)

[bib8] Fagin JA Wells SA. 2016 Biologic and clinical perspectives on thyroid cancer. New England Journal of Medicine 375 2307. (10.1056/NEJMc1613118) 27959677

[bib9] Gudmundsson J Sulem P Gudbjartsson DF Jonasson JG Sigurdsson A Bergthorsson JT He H Blondal T Geller F Jakobsdottir M, *et al*. 2009 Common variants on 9q22.33 and 14q13.3 predispose to thyroid cancer in European populations. Nature Genetics 41 460–464. (10.1038/ng.339) 19198613 PMC3664837

[bib10] Irish Cancer Society. About thyroid cancer. Dublin, Ireland: Irish Cancer Society, 2017. (available at: https://www.cancer.ie/cancer-information/thyroid-cancer/about#sthash.vzvzkEvC.dpbs)

[bib11] Jendrzejewski JP Sworczak K Comiskey DF De La Chapelle A Clinical implications of GWAS variants associated with differentiated thyroid cancer. Endokrynologia Polska 70 423–429. (10.5603/EP.a2019.0027) 31681970

[bib12] Jones AM Howarth KM Martin L Gorman M Mihai R Moss L Auton A Lemon C Mehanna H Mohan H, *et al*. 2012 Thyroid cancer susceptibility polymorphisms: confirmation of loci on chromosomes 9q22 and 14q13, validation of a recessive 8q24 locus and failure to replicate a locus on 5q24. Journal of Medical Genetics 49 158–163. (10.1136/jmedgenet-2011-100586) 22282540 PMC3286794

[bib13] Kang J Deng XZ Fan YB Wu B Relationships of FOXE1 and ATM genetic polymorphisms with papillary thyroid carcinoma risk: a meta-analysis. Tumour Biology 35 7085–7096. (10.1007/s13277-014-1865-5) 24756757

[bib14] Khan A Smellie J Nutting C Harrington K Newbold K Familial nonmedullary thyroid cancer: a review of the genetics. Thyroid 20 795–801. (10.1089/thy.2009.0216) 20465534

[bib15] Liyanarachchi S Wojcicka A Li W Czetwertynska M Stachlewska E Nagy R Hoag K Wen B Ploski R Ringel MD, *et al*. 2013 Cumulative risk impact of five genetic variants associated with papillary thyroid carcinoma. Thyroid 23 1532–1540. (10.1089/thy.2013.0102) 23659773 PMC3868253

[bib16] National Cancer Registry Ireland. Cancer Trends No 16: Cancer of the Thyroid. Cork, Ireland: National Cancer Registry Ireland, 2012. (available at: https://www.ncri.ie/sites/ncri/files/pubs/CancerTrendsNo.16-CancersoftheThyroid_0.pdf)

[bib17] O’Dushlaine CT Morris D Moskvina V Kirov GInternational Schizophrenia Consortium, Gill M Corvin A Wilson JF Cavalleri GL Population structure and genome-wide patterns of variation in Ireland and Britain. European Journal of Human Genetics 18 1248–1254. (10.1038/ejhg.2010.87) PMC2987482 20571510

[bib18] Perros P Boelaert K Colley S Evans C Evans RM Gerrard Ba G Gilbert J Harrison B Johnson SJ Giles TE, *et al*. 2014 Guidelines for the management of thyroid cancer. Clinical Endocrinology 81 1–122. (10.1111/cen.12515) 24989897

[bib19] Poller DN Glaysher S. 2017 Molecular pathology and thyroid FNA. Cytopathology 28 475–481. (10.1111/cyt.12492) 29165888

[bib20] Saenko VA Rogounovitch TI. 2018 Genetic polymorphism predisposing to differentiated thyroid cancer: a review of major findings of the genome-wide association studies. Endocrinology and Metabolism 33 164–174. (10.3803/EnM.2018.33.2.164) 29947173 PMC6021315

[bib21] Tomaz RA Sousa I Silva JG Santos C Teixeira MR Leite V Cavaco BM FOXE1 polymorphisms are associated with familial and sporadic nonmedullary thyroid cancer susceptibility. Clinical Endocrinology 77 926–933. (10.1111/j.1365-2265.2012.04505.x). 22882326

[bib22] Wang F Yan D Ji X Han J Chen M Qiao H Zhang S rs965513 polymorphism as a common risk marker is associated with papillary thyroid cancer. Oncotarget 7 41336–41345. (10.18632/oncotarget.9324) PMC5173063 27191655

[bib23] Xing M Molecular pathogenesis and mechanisms of thyroid cancer. Nature Reviews: Cancer 13 184–199. (10.1038/nrc3431) PMC3791171 23429735

